# Nasopharyngeal carcinoma in children and adolescents - a single institution experience of 158 patients

**DOI:** 10.1186/s13014-014-0274-7

**Published:** 2014-12-05

**Authors:** Weixin Liu, Yuan Tang, Li Gao, Xiaodong Huang, Jingwei Luo, Shiping Zhang, Kai Wang, Yuan Qu, Jianping Xiao, Guozhen Xu, Junlin Yi

**Affiliations:** Department of Radiation Oncology, Cancer Hospital, Chinese Academy of Medical Sciences. No 17, Panjiayuannanli, Chaoyang District Beijing, 100021 China

**Keywords:** Nasopharyngeal carcinoma, Childhood, Adolescence, Radiotherapy, Prognosis

## Abstract

**Background:**

To evaluate the clinical features, treatment results, prognostic factors and late toxicities of nasopharyngeal carcinoma in children and adolescents.

**Methods:**

Between January 1990 and January 2011, 158 NPC patients younger than 20 years old were treated in our institution, and the patient’s clinical characteristics, treatment modalities, outcomes and prognostic factors were retrospectively analyzed.

**Results:**

There were 9 (5.7%) patients in stage II, 60 (38.0%) in stage III and 89 (56.3%) in stage IV according to the UICC2002 staging system. Neck mass (32.3%), headache (21.5%) and nasal obstruction (15.2%) were the most common chief complaints. With a median follow-up time of 62.5 months (range 2.0-225.0 months), the 5-year overall survival (OS) rate, local-regional control (LRC) rate and distant metastasis-free survival (DMFS) rate were 82.6%, 94.9% and 76.4%, respectively. There were 43 (27.2%) patients failed during the follow up, with seven local-regional recurrences and 38 distant metastases. In univariate analysis, the 5-year OS of T4 and T1-3 were 75% and 87.9%, *p* = 0.01, stage IV and stage II-III were 77.1% and 90%, *p* = 0.04, respectively. In multivariate analysis, T4 (*p* = 0.02) and stage IV (*p* = 0.04) were the independent adverse prognostic factors for OS. Significant reduction in trismus (27.3% *v* 3.6%, *p* = 0.03) and G2 xerostomia (37.9% *v* 10.3%, *p* = 0.02) was observed in patients treated by IMRT.

**Conclusions:**

Most childhood and adolescence nasopharyngeal carcinoma patients were locally advanced diseases at first diagnosed. The treatment results of radiotherapy, with or without chemotherapy, are excellent in our institution. Reducing distant metastasis with new strategies and late toxicities with intensity-modulated radiotherapy are the future directions for the treatment of adolescent nasopharyngeal carcinoma.

## Background

Nasopharyngeal carcinoma (NPC) is the most commonly diagnosed head and neck malignancy in China and Southeast Asian countries, but children and adolescent nasopharyngeal carcinoma is very rare worldwide. The incidence among children and adolescents varies greatly among different regions and races, accounts for 0.1-2.3% of all NPCs in our nation [1,2] and 2%-18% in other countries [3-5]. Standard therapy for NPC in children and adolescents has generally followed the guidelines established for adults. External beam radiotherapy has been the mainstay of such treatment, and concurrent chemoradiotherapy has been confirmed to be effective in adult patients [4]. The published results of NPC in the young are diverse, most involving a small number of patients, non-uniform regimens both in radiotherapy and chemotherapy. Although childhood and adolescence NPC patients are usually associated with advanced local-regional disease at first diagnoses and a highly prevalence of distant metastasis, the outcomes are generally better than adult NPC [5,6]. In this paper, we collected a large sample size with relative homogenous treatment to evaluate the clinical characteristics, treatment results, prognostic factors and late toxicities. We hope to provide further information on the treatment of childhood and adolescence nasopharyngeal carcinoma in the future.

## Methods

### Patient population

Between January 1, 1990 and January 31, 2011, 168 pathologically confirmed NPC patients under 20 years old were treated in our institution. Ten patients were excluded due to their undefined stage or lost follow-up shortly after treatment. The median age was 16 years (range 8–20 years). All patients received thorough physical examinations, biopsies of the nasopharynx, dental care, general status evaluations, blood counts, chest X-rays, as well as neck and abdomen ultrasonography. Indirect mirrors (n = 158,100%) and fiberoptic nasopharyngoscopes (n = 72, 45.6%) were used to examine the nasopharynx. All patients were restaged according to the UICC 2002 staging system. 153 patients (96.8%) were staged with either CT, MRI or both, among them, 76 patients (48.1%) had bone scans before treatment. X-rays and clinical examinations were used in the other five as staging tools.

### Treatment

All patients received radical external beam radiotherapy, there were 24 (15.2%) patients received neoadjuvant chemotherapy, 43 (27.2%) patients in stage III/IV received concurrent chemo-radiotherapy and 20 (12.7%) patients with residue primary lesions after radiotherapy received adjuvant chemotherapy.

### Radiotherapy

There were 103 (65.2%) patients received conventional 2D radiotherapy using 6 MV photons combined with 6–12 MeV electrons. The technique and dose used were as follows: first, a facial-cervical field (encompassing the nasopharynx, paranasopharyngeal fissure, skull base, pterygopalatine fossa, one third to one half of the posterior nasal cavity and maxillary sinus, and the upper neck lymph drainage region; usually the inferior margin of the field was set at the lower edge of hyoid bone) to a total dose of 36 Gy, followed by a smaller facial-cervical field (when the tumor involved the oropharynx or retropharyngeal lymph node) or a preauricular field to avoid excessive exposure of the brainstem and the spinal cord, to a total dose of 70–72 Gy to the nasopharyngeal region. The irradiation dose to the neck lymph node drainage region was as follows: 50 Gy was delivered to the lower neck and supraclavicular region and 60 Gy to the upper neck when cervical nodes were negative. 60 Gy were applied to the entire neck and supraclavicular region when cervical nodes were positive, then 70–72 Gy to the positive lymph nodes, with the division of the upper and lower neck set at the lower edge of the cricoid bone. After 70–72 Gy of irradiation, an additional of 10–21 Gy was boosted to the residue tumor with a shrinking-field external beam, brachytherapy or stereotactic radiosurgery technique. The residual lymph nodes were removed by surgical excision or regional neck dissection after 2–3 months observation.

There were 55 (34.8%) patients received intensity-modulated radiation therapy (IMRT) technique. The entire neck IMRT technique was used to cover the primary lesion, nodal disease and entire neck including the supraclavicular region. The prescription dose for T1 and T2 primary lesions (GTVp) was 70 Gy in 33 fractions at 2.12 Gy per fraction, while 74 Gy at 2.24 Gy per fraction was applied to T3 or T4 diseases and involved retropharyngeal nodes with a maximum diameter > 1.5 cm, and all positive lymph nodes (GTVnd) received 70 Gy at 2.12 Gy per fraction. The elective radiation dose of 60 Gy at 1.82 Gy per fraction encompassed the high-risk regions including the uninvolved skull base, parapharyngeal spaces, the posterior third of the nasal cavity and high-risk nodal levels. If there were no positive neck nodes in the neck, 50–54 Gy was delivered to the bilateral lower neck and supraclavicular region using a two-phase IMRT plan, with the phase 1 IMRT plan (28 treatment fractions) covering the primary lesion, positive nodes, the high-risk region and the lower neck/supraclavicular region. The phase 2 IMRT plan (5 treatment fractions) covered only the primary lesion, positive nodes and high-risk regions. The dose limited to the major organs at risk were as follows: the brain stem with a 3 mm margin, Dmax < 54 Gy; spinal cord with a 5 mm margin, Dmax < 40 Gy; the optic nerve, chiasm and temporal lobe, Dmax < 54 Gy; and the parotid gland, V30-35 < 50%.

For patients who received neoadjuvant chemotherapy, the target volumes depended on the pre-chemo involved regions.

### Chemotherapy

Seventy (44.3%) patients received chemotherapy, with 24, 43 and 20 patients treated by neoadjuvant, concurrent and adjuvant chemotherapy, respectively. Neoadjuvant chemotherapy consisted of cisplatin and a 5-FU based regimen(PF regimen) every 3 weeks for 1–2 cycles. In concurrent chemotherapy, 34 patients received a cisplatin 30 mg/m^2^/week regimen (median cycles: 7), 9 patients received cisplatin 80–100 mg/m^2^/q3w regimen (median cycles: 3), and adjuvant chemotherapy were PF-based regimen for 2–6 cycles.

### Intra- and Post- treatment assessments

All patients were evaluated weekly during radiation therapy, with a required follow-up after they completed radiotherapy: one month after the completion of radiotherapy, every three months in the first two years, every six months from the second to fifth years, and annually thereafter. Late radiation effects were evaluated and scored according to the Radiation Therapy Oncology Group/European Organization for Research and Treatment of Cancer late effects scale.

### Statistical analysis

The statistic was performed by SPSS 19.0 software. Kaplan-Meier method was used for calculating the survival, chi-square test and Cox regression analysis were used to detect statistically significant differences among the late toxicities and potential prognostic factors between the different groups.

### Ethical statement

This study has been approved by ethics committee of cancer hospital, Chinese academy of Medical Sciences.

## Results

### Patients’ clinicopathological characteristics

Among 158 patients eligible for analysis, there were 119 male and 39 female, with median age of 16 years old (range 8–20 years), 9 patients in stage II, 60 in stage III and 89 in stage IV, respectively. 139 (88%) patients were non-keratinizing diseases. The median history time was 4.8 months (range 0.2-60.0 months). Neck mass (32.3%), headache (21.5%) and nasal obstruction (15.2%) were the most common chief complaints. There were 36 patients (22.8%) observed cranial nerve palsy, the grigeminal nerve (V) and abducent nerve (VI) were the most commonly involved, with incidence of 7.6% in V1 (12), 12.0% in V2 (19), 10.1% in V3 (16) and 6.3% in VI repectively (Table 1).

**Table 1:** **The characteristics of patients**

**Characteristic**	**Number n (%)**
Gender	
Male	119(75.3)
Female	39(24.7)
Age	
<16	86(54.4)
≥16	72(45.6)
Symptom/sign	
Neck mass	115(72.8)
Headache	94(59.5)
Tinnitus	82(51.9)
Blood-tinged drainage	76(48.1)
Hearing loss	70(44.3)
Nasal obstruction	63(39.9)
Cranial nerve palsy	36(22.8)
Other	17(10.8)
Diplopia	14(8.9)
Facial anesthesia	12(7.6)
Histopathology	
Moderately differentiated SCC	3(1.9)
Poor differentiated SCC	139(88)
Undifferentiated SCC	1(0.6)
Other	15(9.5)
T classification	
T1/T2/T3/T4	4/34/56/64(2.5/21.5/35.4/40.5)
N classification	
N0/N1/N2/N3	10/32/83/33(6.3/20.3/52.5/20.9)
Stage	
II/III/IV	9/60/89(5.7/38.0/56.3)
Irradiation technique	
2D and CRT	103(65.2)
IMRT	55(34.8)
Treatment	
Radiotherapy alone	85(53.8)
Combined modality therapy	73(46.2)

### Survival and prognostic factors

**Figure 1:**
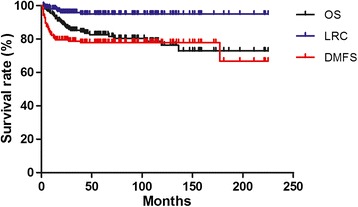
**Kaplan-Meier survival curves of 158 children and adolescents nasopharyngeal carcinoma.** The 5-year overall survival (OS), local-regional control (LRC) and distant metastasis free survival (DMFS) were 82.6%, 94.9% and 76.4%, respectively.

The median follow-up time was 62.5 months (range 2.0-225.0 months), with 13.3% patients lost follow-up. The 5-year OS, LRC and DMFS were 82.6%, 94.9% and 76.4%, respectively (Figure 1). In univariate analysis (Table 2), the 5-year OS of T4 and T1-3 were 75% and 87.9%, *p* = 0.01, stage IV and stage II-III were 77.1% and 90%, *p* = 0.04, respectively. In multivariate analysis, T4 (*p* = 0.02) and stage IV (*p* = 0.04) were the independent adverse prognostic factors for OS. No significant prognostic factors were found for LRC and DMFS in either univariate or multivariate analysis.

**Table 2:** **Univariate analysis (log-rank test)**

**Factors**	**5-y OS %**	***P*** **value**	**5-y LRC %**	***P*** **value**	**5-y DMFS %**	***P*** **value**
Gender		0.43		0.48		0.07
Male	81.1		93.9		73.0	
Female	86.6		97.4		86.6	
Age		0.17		0.54		0.76
≤16	78.4		95.6		77.2	
>16	87.5		94.0		75.5	
History		0.45		0.98		0.92
≤6 m	80.5		94.7		77.1	
>6 m	87.7		95.2		74.7	
UICC-T						
		0.80		0.67		0.99
T1	66.7		100		75.0	
T2-4	82.9		94.7		76.5	
		0.34		0.77		0.64
T1-2	83.3		93.1		78.7	
T3-4	82.3		95.4		75.7	
		0.01		0.32		0.54
T1-3	87.9		96.2		76.9	
T4	75.0		92.9		75.7	
UICC-N						
		0.95		0.48		0.30
N0	77.1		100		90.0	
N1-3	82.9		94.5		75.4	
		0.48		0.10		0.19
N0-1	89.6		100		83.3	
N2-3	79.9		92.8		73.8	
		0.51		0.61		0.45
N0-2	83.8		95.6		75.2	
N3	77.8		92.6		81.2	
UICC stage						
		0.57		0.53		0.36
II	85.7		100		88.9	
III-IV	82.4		94.6		75.7	
		0.04		0.10		0.87
II-III	90.0		98.4		75.9	
IV	77.1		92.0		76.9	
Treatment		0.54		0.36		0.57
Radiotherapy alone	82.9		93.0		77.8	
Modality therapy	82.7		97.2		75.0	
Neoadjuvant chemotherapy		0.70		0.25		0.85
Yes	87.3		100		78.6	
No	80.8		93.9		76.1	
Concurrent chemotherapy		0.17		0.58		0.71
Yes	85.3		97.6		78.8	
No	67.3		100		75.5	
Adjuvant chemotherapy		0.30		0.27		0.68
Yes	90.0		89.7		80.0	
No	72.9		100		75.9	
Technology		0.33		0.74		0.42
2D	79.5		94.2		74.0	
IMRT	88.7		96.1		81.1	
Dose		0.45		0.45		0.63
>70 Gy	86.8		93.7		74.2	
≤70 Gy	77.1		96.6		79.4	
Response		0.11		0.21		0.46
PR	75.2		92.1		74.5	
CR	89.2		97.2		79.4	

### Failure pattern

There were 43 patients (27.2%) failed during follow up, with 7 in local-regional, 38 in distant metastasis and 2 in both. The median failure times were 15 months (3–39 months) and 5 months (1–38 months) for local-regional recurrence and distant metastasis. The most common site of metastasis was bone (27 patients, 17.1%); other sites included lung (n = 7), liver (n = 1) and distant lymph nodes (n = 3). The causes of death were primary disease in 24, second malignancy in 2 and unknown in 4.

### Late toxicities

For those patients who had detail medical records, the most common late toxicities were xerostomia, neck fibrosis, and hearing loss, with incidence of 94.8% (55/58), 91.2% (52/57) and 52.6% (30/57) respectively. Hypothyroidism was reported in 50.8% (30/59) patients. Severe late sequela included blindness (2 patients), deafness (2 patients), bone necrosis (2 ptatients) and nasopharynx hemorrhage (1 patient). Due to a lack of sufficient information, radiation encephalopathy could not be fully documented (see detail in Table 3). When compared to the patients treated by 2D conventional technique, significant reduction in trismus (27.3% *v* 3.6%, *p* = 0.03) and G2 xerostomia (37.9% *v* 10.3%, *p* = 0.02) was observed in those patients treated by IMRT.

**Table 3:** **Late toxicities**

	**Technique**	**Grade 0 (%)**	**Grade 1 (%)**	**Grade 2 (%)**	**Grade 3 (%)**	**Grade 4 (%)**	**p value**
Skin (n = 54)	IMRT(19)	3(15.8)	14(73.7)	2(10.5)	0	0	0.377
	2D(35)	12(34.3)	18(51.4)	4(11.4)	1(2.9)	0	
Mucosa (n = 55)	IMRT(20)	14(70.0)	5(25.0)	1(5.0)	0	0	0.152
	2D(35)	15(42.9)	17(48.6)	3(8.6)	0	0	
Neck fibrosis (n = 57)	IMRT(21)	3(14.3)	8(38.1)	8(38.1)	2(9.5)	0	0.672
	2D(36)	2(5.6)	15(41.7)	12(33.3)	6(16.7)	1(2.8)	
Xerostomia (n = 58)	IMRT(21)	1(4.7)	14(66.7)	6(28.6)	0	0	0.063
	2D(37)	2(5.4)	13(35.1)	22(59.5)	0	0	
Spinal cord (n = 49)	IMRT(18)	18(100)	0	0	0	0	0.441
	2D(31)	30(96.8)	1(3.2)	0	0	0	
Hearing (n = 57)	IMRT(20)	10(50.0)	0	10(50.0)	0	0	0.568
	2D(37)	17(45.9)	0	18(48.6)	0	2(5.4)	
Vision (n = 53)	IMRT(18)	17(94.4)	1(5.6)	0	0	0	0.530
	2D(35)	32(91.4)	1(2.9)	0	0	2(5.7)	
Trismus (n = 55)	IMRT(18)	16(88.9)	0	1(5.6)	1(5.6)	0	0.091
	2D(37)	22(59.5)	0	12(32.4)	1(2.7)	2(5.4)	
Hypothyroidism (n = 59)	IMRT(39)	19(48.7)	0	20(51.3)	0	0	0.926
	2D(20)	10(50.0)	0	10(50.0)	0	0	

There were six patients (3.8%) developed second primary neoplasm in the radiation field after 38–123 months of radiotherapy (Table 4).

**Table 4:** **Second primary neoplasm**

**Gender**	**Age**	**Onset year**	**Stage**	**Treatment**	**Technique**	**Primary dose**	**Neck dose**	**Month after treatment**	**Site**	**Pathology**
M	17	1992	T3N2M0	RT	2D	72.5 + 10^※^	80/62	116	Lower jaw bone	NA
F	16	2000	T2N2M0	RT + CT	2D	70 + 6^※^	70/50	48	Neck	Fibrosarcoma
M	11	1994	T4N3M0	RT	2D	74	66/52	119	Maxillary sinus	Fibrosarcoma
M	16	1995	T2N3M0	RT	2D	70 + 16^※^	70/70	37	Soft palate	Papilloma
F	12	1998	T4N2M0	RT	2D	70	70/60	153	Cervical vertebra	NA
M	16	1996	T4N1M0	RT	2D	70	70/60	101	Upper jaw bone	Chondrosarcoma

^※^was boosted by SRS or IMRT or cone down field.

## Discussion

The incidence of NPC varies widely among different regions and races, and is most often seen among patients in their fifth or sixth decade in endemic countries. However, there was a bimodal incidence graphs in sporadic regions such as North America and Mediterranean basin countries, a second minor early peak was observed at 10–20 years of age, with incidence of 2-18% [7]. Huang et al. [1] reported the number of patients younger than 15 years old was 53 (0.1%) among 54,304 NPC patients in China. Our group accounted for 1.33% of all NPC patients from 1990–2011 (total 3,081 patients). Similar to adults, the children and adolescents with NPC in our study usually presented with neck masses, headaches, tinnitus and cranial nerve palsy. On the other hand, children and adolescents patients had a higher proportion of non-keratinization carcinoma (88%) and advanced diseases (stage III-IV, 94.3%). This result was similar to other reports, with non-keratinizing carcinoma accounted for 71.4%-96.4% of all patients, and stage III-IV patients constituted 78.5%-96% [6-12]. In our study, the 5-year OS, LRC and DMFS of 82.6%, 94.9% and 76.4%, respectively, were higher than in other studies with a 5-year OS ranging from 49%-79% and a 5-year DFS varying from 47%-73% [2,4,6,8,9,11-14]. The higher survival rate may result from higher radical radiation dose and a boost dose to the residue lesions after radical radiation dose [15,16]. Several studies found that children and adolescent NPC patients have better results compared with adult NPC patients, the 5 years OS were 71% v 58% (p = 0.03) [6] and the 5 years disease-specific survival were 83% ± 3.9% *v* 62% ± 0.8% (p < 0.001) [5].

In our univariate and multivariate analysis, T4 was the adverse prognostic factor. T4 patients usually have a larger tumor volume than those with T1-3 and a higher tumor burden needs a higher dose to control. Sze et al. [17] found that the correlation between T stage and tumor volume was a highly significant factor among 308 nasopharyngeal carcinoma patients, and the risk of local failure was estimated to increase by 1% for every 1 cm^3^ increase in primary tumor volume. Although the recommended dose for the primary site ranges from 59.4 Gy to 66 Gy (1.8-2.0 Gy per fraction) in the literature, it seems that a higher dose provided better outcomes; Ozyar et al. [8] reported that dose > 66 Gy had promising LRRFS (*p* = 0.01) in multivariate analysis, while other authors reported that a 64-80Gy or higher dose was needed [2,18]. In contrast, Hu et al. [13] reported that higher radiation dose (> 70 Gy) did not promise better local control or survival, and some authors believed that higher dose would inevitably damage normal tissue, causing a high incidence of severe late sequelae and second malignancies [7,10]. In our study, the incidence of common late complications, such as xerostomia, neck fibrosis, hearing loss and trismus, were similar to or slightly lower than in other reports [2,10,13,14]. Wolden et al. [10] found that neither the addition of chemotherapy nor the radiation dose was statistically predictive for adverse sequelae. However, there was a slight trend toward fewer severe complications when 3D conformal radiotherapy and IMRT were used compared with 2D conventional radiation techniques (0% *v* 28%). Laskar et al. [19] reported a significant reduction in the incidence of acute Grade 3 toxicity and a considerable prolongation in the median time to the onset of Grade 2 toxicity with IMRT. Our study showed consistent findings of prevention in late toxicity by using IMRT. The use of IMRT resulted in a significant reduction of trismus (*p* = 0.03) and G2 xerostomia (*p* = 0.02) because of IMRT allows for the delivery of high doses to the target area while sparing the surrounding critical structures and offers superior target coverage compared with conventional radiotherapy and 3D-CRT in improving therapeutic ratios. Using new techniques, such as IMRT, to improve local control and protect normal tissue will be a key focus in future daily practice.

Many studies focused on using neoadjuvant chemotherapy combined with lower dose radiotherapy to reduce the radiation related severe late toxicities. In NPC-91-GPOH study and NPC-2003-GPOH/DCOG study [20,21], after combination of chemotherapy and radiation (54–59.4 Gy) followed by INF-β-1a, complete remesion was accomplished in 58/59 and 43/45 of patients, and excellent disease-free survival and overall survival were achieved in advanced NPC patients. In Orbach’s retrospective study, the 5 year OS and EFS of patients who had a good response to neoadjuvant chemotherapy with a low radiation dose (≤50 Gy) were 74% and 76%, so the author proposed that a reduction radiation dose to chemo-sensitive patients was feasible [22].

In the present series of young NPC patients, distant metastasis remained the major pattern of failure [2,12,20],which indicated that subclinical metastasis had already existed at first treatment, requiring early systemic therapy. However, the most effective chemotherapy regimens and their optimal timing with radiation therapy remained to be determined. Due to the rare incidence rate of children and adolescent nasopharyngeal carcinoma, multi-center collaboration on RCTs is needed to establish the treatment guidelines for pediatric NPCs.

## Conclusion

Most childhood and adolescence nasopharyngeal patients had local advanced diseases at first diagnosed. The treatment results of radiotherapy with or without chemotherapy are excellent in our institution. Reducing distant metastasis with new strategies and late toxicities with intensity-modulated radiotherapy will be the future directions for the treatment of children and adolescent nasopharyngeal carcinoma. Further, response-adapted RT is worth further evaluation to minimize late toxicities.

## Abbreviations

NPC: Nasopharyngeal carcinoma
IMRT: Intensity-modulated radiation therapy
GTVp: Gross tumor volume (primary)
GTVnd: Gross tumor volume (lymph node)
LRC: Local-regional control
DMFS: Distant metastasis free survival
OS: Overall survival
3D-CRT: 3 dimensional- conformal radiation therapy
